# Research on the Tribological Behavior of Polyurethane Acrylate Coatings with Different Matrix Constituents as Well as Graphite and PTFE

**DOI:** 10.3390/polym17081121

**Published:** 2025-04-21

**Authors:** Weihua Cao, Xiao Yang, Zhenjie Song, Jia Geng, Changxin Liu, Ning Zhang, Xiaowen Qi

**Affiliations:** 1 School of Mechanical Engineering, Yanshan University, Qinhuangdao 066004, China; caoweihua.ysu@foxmail.com (W.C.); szj_ysu@163.com (Z.S.); sittihel@stumail.ysu.edu.cn (J.G.); cxliu@ysu.edu.cn (C.L.); 2 Key Laboratory of Self-Lubricating Spherical Plain Bearing Technology of Hebei Province, Yanshan University, Qinhuangdao 066004, China; 3 Department of Economics and Management, Hebei University of Environmental Engineering, Qinhuangdao 066102, China; zhangn202410@163.com

**Keywords:** polyurethane acrylate, coatings, solid lubricant, monomers, wear resistance

## Abstract

With the aim of developing a wear-resistant ultraviolet (UV)-cured self-lubricating coating, this study investigated the impact of matrix components and lubricants on UV-cured interpenetrating polymer network-polyurethane acrylate (IPN-PUA) self-lubricating coatings. Four coatings with different monomer combinations were prepared, using isophorone diisocyanate (IPDI) or tolylene-2,4-diisocyanate (TDI) in combination with hydroxypropyl acrylate (HPA) or 2-hydroxyethyl acrylate (HEA). These coatings were denoted as IPDI-HPA, IPDI-HEA, TDI-HPA, and TDI-HEA, respectively. The surface morphologies, compositions, friction and wear properties, as well as the comprehensive performances were investigated. The results indicated that IPDI-HPA had the lowest surface roughness and that TDI-HEA had the smallest wear rate, while TDI-HPA showed the best overall performance (roughness of 1.485 μm, coefficient of friction (COF) of 0.746, and wear rate of 10.64 × 10^−14^ m^3^/N·m). With TDI-HPA as the matrix, graphite and polytetrafluoroethylene (PTFE) particles of different sizes were added as lubricants. The T-P-25F (TDI-HPA coating with 25 μm sized PTFE) coating had self-lubricating capabilities, as was manifested by a friction coefficient of 0.395, which was 47% lower than that of the pure TDI-HPA coating, and it simultaneously showed outstanding wear-resistance performance. The wear rate of the T-P-25F coating was 3.97 × 10^−14^ m^3^/N·m, 62.7% lower than that of the pure TDI-HPA coating. This research provides valuable guidance for optimizing the performance of such coatings and yields a self-lubricating coating with excellent wear resistance.

## 1. Introduction

During the operation of mechanical components, friction and wear take place between the connecting parts. Such friction and wear consumes a considerable amount of energy and leads to equipment damage. Self-lubricating polymer coatings, owing to their characteristics such as low density and easy moldability, are widely employed in the mechanical components of critical equipment and industrial production fields including aerospace, engineering machinery, rail transit, petrochemical metallurgy, and energy and power [1,2]. However, in comparison with other hard coatings, polymer coatings exhibit relatively poor wear resistance. Hence, the development of a wear-resistant coating is of vital importance for reducing equipment failures resulting from wear.

The tribological properties of polymer coatings have been widely studied. Wang et al. [3] investigated coatings with polyamide-imide, polyimide, and phenolic epoxy resin as the matrix; graphene oxide (GO), graphite, polytetrafluoroethylene (PTFE), and WS_2_ as lubricants; and SiC and ZrO_2_ as additives. When the content of WS_2_ was 7.5 wt.%, the COF of the composite coating reached 0.2, and the wear rate reached 3.4 × 10^−14^ m^3^/N·m. Fernández-Álvarez et al. [4] studied the tribological properties of epoxy powder coatings modified with SiO_2_ nanoparticles and found that the COF finally stabilized between 0.7 and 0.8, and the wear rate reached 5.3 × 10^−13^ m^3^/N·m. Chen et al. [5] improved the tribological properties of epoxy resin by using micro-arc oxidation-modified graphene, with a COF of 0.15 and a wear rate of 6.17 × 10^−14^ m^3^/N·m. Zhang et al. [6] investigated the tribological properties of a polyurethane composite coating with MoS_2_-multi-walled carbon nanotube hybrids. The COF of the coating reached 0.1, and the wear rate reached 4 × 10^−14^ m^3^/N·m. Bai et al. [7] studied the tribological properties of the aligned GO@Fe_3_O_4_/WPU composite coating, with a COF of 0.2 and a wear rate of 3 × 10^−13^ m^3^/N·m. Tang et al. [8] prepared a superhydrophobic polyurethane/MoS_2_ nanocomposite coating with wear resistance with a COF of 0.38. The wear life of this coating reached more than 6000 friction cycles, showing good wear resistance. Ching et al. [9] studied the tribological properties of an acrylic-based polyurethane coating filled with nano-SiO_2_, with a COF of 0.3 and a wear rate of 3 × 10^−15^ m^3^/N·m. Although the composite coatings have achieved relatively ideal friction coefficients, their wear rates are still relatively high. Therefore, it is necessary to further improve the mechanical properties of the coatings to obtain more wear-resistant polymer-based coatings.

An interpenetrating polymer network (IPN) [10,11,12] represents a distinctive intertwined polymer network architecture characterized by multiphase and multicomponent natures. The IPN technology enables the realization of physical blending through chemical approaches. Benefiting from this, IPN technology has wide applications in hydrogels [13,14,15,16], damping materials [17,18,19,20], adhesives [21,22,23], etc. Due to the advantages of IPN technology, it is widely used in coatings [24,25,26]. The monomers that construct the network structure exert an impact on the mechanical properties of IPN-structured polymer coatings. Tavsanli et al. [27] studied IIR/PC18A (butyl rubber/poly (n-octadecyl acrylate)) IPN coatings. It was discovered that the grafted IIR could enhance the chemical cross-link density of IPNs approximately tenfold. Acrylate-functionalized IIR exhibited the highest modulus and fracture stress, which could be attributed to the varying relative contributions of the chemical and physical cross-links. Carbajo-Gordillo et al. [28] performed an in-depth exploration of PHU (polyhydroxyurethane)/PVA or PHU/gelatin semi-IPN hydrogel coatings. The influences of 1-thioglycerol and 1,2-dithioglycerol monomers on the properties of PHU/PVA coatings were examined. It was found that the addition of 1,2-dithioglycerol led to a remarkable improvement in elasticity, as manifested by the notably lower loss tangent values. These research findings indicate that the variety of monomers is crucial for boosting the mechanical properties and further enhancing the wear-resistance capabilities in the coating system. Nevertheless, how monomer structure impacts the tribological behaviors of coatings demands more in-depth scrutiny.

Since IPN-structured polymers do not inherently possess self-lubricating properties, it becomes extremely crucial to enhance their self-lubricating performance when deployed in the field of tribology. Chen et al., Yu et al., and Xia et al. respectively used polydimethylsiloxane [29], SiC submicron particles and short carbon fibers [30], and graphene oxide [31] and nanodiamonds [32] to conduct tribological modifications on polyurethane/epoxy resin IPN composites and obtained self-lubricating composites with both excellent tribological properties and good damping performance. Yu et al. [33] prepared self-lubricating materials with favorable mechanical properties by regulating the microstructure of the polyurethane/epoxy resin interpenetrating network structure, which exhibited good self-lubricating and wear-resistant properties under water lubrication and boundary lubrication conditions. Lin et al. [34] used Si_3_N_4_@MoS_2_ to enhance the wear resistance of epoxy resin/polyacrylate IPN composite coatings. Nevertheless, research on the tribological properties of IPN-structured coatings with different particle sizes and types of solid lubricants is still relatively scarce. Li et al. [35] probed into how various PTFE particle sizes affected the tribological characteristics of epoxy resin-based bulk self-lubricating substances. They discovered that larger-sized PTFE particles were more effective in enhancing the friction performance of these materials. Ceylan et al. [36] investigated the influence of adding different solid lubricants of PTFE and graphite on the friction properties of rigid and toughened epoxy resins. It was found that whether in rigid epoxy or toughened epoxy, graphite has an advantage over PTFE in improving the friction performance of epoxy in terms of COF reduction. However, due to the differences in the molding methods and prepolymer viscosities between bulk materials and coating materials, whether these influence mechanisms are applicable to coating materials still requires further research.

Concerning the production of polymer coatings, classic preparation means include brushing, rolling, dipping, spin-coating, and spraying. All of these methods necessitate dissolving the polymer in the corresponding volatile solvents followed by its application onto the substrate. After the solvent evaporates, a coating is formed [37,38,39,40]. These methods are characterized by low efficiency. Moreover, the solvent can cause environmental pollution and necessitates mandatory recovery, resulting in high costs. Ultraviolet (UV) curing technology, whose molding process does not require a solvent, has the characteristics of high efficiency, environmental protection, energy conservation, and excellent coating properties, making it widely used in recent years [41]. In previous research [42], we successfully prepared IPN-PUA self-lubricating bulk composites through a thermal curing method. The use of a photoinitiator endows the material with the potential for photocuring molding. However, research on the influence of the types of monomers and solid lubricants on material properties has not been conducted yet.

This research explored the influence of matrix constituents and lubricants on the properties of UV-cured polyurethane acrylate (PUA) self-lubricating coatings with an interpenetrating polymer network (IPN) structure and acquired a self-lubricating coating with excellent wear resistance. By selecting monomers to form IPN-structured polymers of diverse compositions, an in-depth investigation was conducted on the surface roughness, tribological properties, and wear morphology of the coatings. It was found that both isocyanate with a benzene ring structure and short-chain hydroxyl acrylate, when used as the monomers of IPN-PUA, could enhance the tribological performance but negatively affect the surface roughness of the coatings. After modifying the tribological properties using two PTFE particles of different sizes along with graphite, experimental evidence demonstrated that within the IPN-PUA coating, the one filled with small-sized PTFE showed the lowest coefficient of friction (COF) and wear rate. This indicates that the manner in which the solid lubricant particle size affects the tribological characteristics of composite materials displays marked differences between bulk and coating materials. This research provides an essential understanding of the factors that influence the self-lubricating performance of UV-cured polyurethane acrylate coatings.

## 2. Materials and Methods

### 2.1. Reagents

All reagents underwent dehydration treatment with a 4 Å molecular sieve prior to the preparation. The commercial sources and specifications of these reagents were as follows: isophorone diisocyanate (IPDI, with a purity of 99%), dibutyltin dilaurate (DBTDL, with a purity of 90%), and hydroxypropyl acrylate (HPA, with a purity of 97%) were all supplied by Meryer Chemical Technology Co., Ltd. (Shanghai, China); tolylene-2,4-diisocyanate (TDI, with a purity of 98%) was provided by Mreda Chemical and Biological Reagents Co., Ltd. (Beijing, China); 2-hydroxyethyl acrylate (HEA, with a purity of 99%) was furnished by Shanghai Macklin Biochemical Technology Co., Ltd. (Shanghai, China); methyl methacrylate (MMA, of analytical pure grade) was supplied by Tianjin Damao Chemical Reagent Factory (Tianjin, China); and 2-hydroxy-2-methylpropiophenone (photoinitiator 1173, with a purity of 99%) was provided by Bide Pharmatech Ltd (Shanghai, China). Additionally, industrial grade graphene oxide (GO) was acquired from Suzhou Tanfeng Graphene Technology Co., Ltd. (Suzhou, China) and was directly used without any further purification.

The solid lubricants were thoroughly dried in a 60 °C constant temperature oven for 12 h before use. Graphite with 1500 mesh was purchased from Dongguan Tangxia Xiangyang Graphite Products Processing Factory (Dongguan, China). The 25 μm sized PTFE particles were obtained via Shanghai Aladdin Biochemical Technology Co., Ltd. (Shanghai, China), while the PTFE particles with a size of 75–140 μm (75 μm sized PTFE particles for simplicity) were secured from Dongguan Xingwang Plastic Material Company (Dongguan, China).

### 2.2. The Procedure for Paint Preparation

The preparation procedures for the four coating paints were consistent and resembled those adopted in our previous work [42].

Initially, 0.1 g of GO, 0.0425 mol of either IPDI or TDI, 10 g of MMA (0.1 mol), and 0.1 g of the catalyst DBTDL were introduced into a three-necked flask. The reaction was conducted in a water bath with the assistance of a magnetic stirrer for a duration of 4 h within a dry nitrogen atmosphere and at a constant temperature of 40 °C. Subsequently, 0.0426 mol of either HPA or HEA was dripped into the flask, after which the temperature was elevated to 70 °C for 4 h. Next, 0.0425 mol of either HPA or HEA was added into the three-necked flask to procure the paints, which were respectively designated as IPDI-HPA, IPDI-HEA, TDI-HPA, and TDI-HEA. During the reaction process, GO initially reacted with IPDI or TDI and subsequently reacted with HPA or HEA [43].

### 2.3. Process of Coating Curing

**Figure 1:**
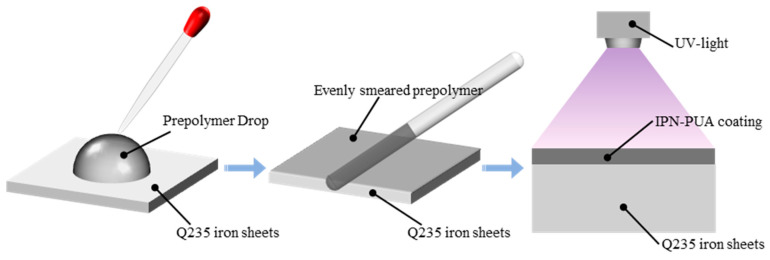
Diagram of the UV curing process of coatings.

Figure 1 clearly demonstrates the UV curing procedure of the coating. At first, 3 wt.% of photoinitiator 1173 was incorporated into the paints and thoroughly blended. Subsequently, Q235 iron sheets (measuring 30 mm × 30 mm × 2.5 mm) were abraded with sandpaper to a 1000-mesh finish and then ultrasonically cleaned with alcohol. Next, 0.5 g of the paint was evenly smeared onto the surface of the iron sheets using a glass rod. Subsequently, the samples were horizontally positioned in a light-shielded environment and allowed to stand for 10 min to ensure the coating achieved a smooth, level finish. Finally, the curing reaction was conducted in a UV curing chamber (possessing a wavelength of 365 nm and a power of 1 kW) for 60 s to fabricate the IPN-PUA coatings. All the coatings were cleansed with ethanol prior to testing.

**Figure 2:**
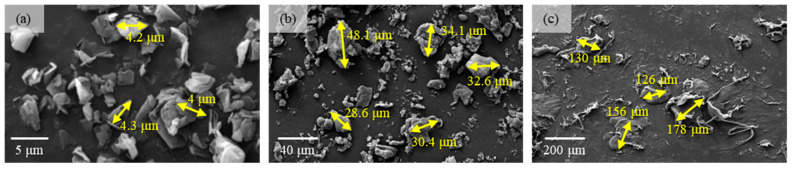
The particle morphologies of solid lubricants. (**a**) Graphite, (**b**) 25 μm sized PTFE particles, and (**c**) 75 μm sized PTFE particles.

For the purpose of improving the tribological performance, solid lubricants were incorporated into the coatings, specifically graphite, 25 μm sized PTFE particles, and 75 μm sized PTFE particles. The particle sizes of these solid lubricants are illustrated in Figure 2. Notably, graphite possesses the smallest particle size of approximately 5 μm.

The molding process of the self-lubricating coatings was identical to that of the pure IPN-PUA coating. Prior to applying the paints onto the iron sheets, the solid lubricants and the paints were thoroughly mixed at a mass ratio of 1:10.

### 2.4. Characterization of Tribological Properties and Surface Morphologies

The infrared spectrum of the coating was scanned by attenuated total reflectance infrared spectroscopy under ambient temperature, with the measurement range spanning from 500 to 4000 cm^−1^ (Nicolet IS5, Thermo Fisher Co., Massachusetts, USA).

**Figure 3:**
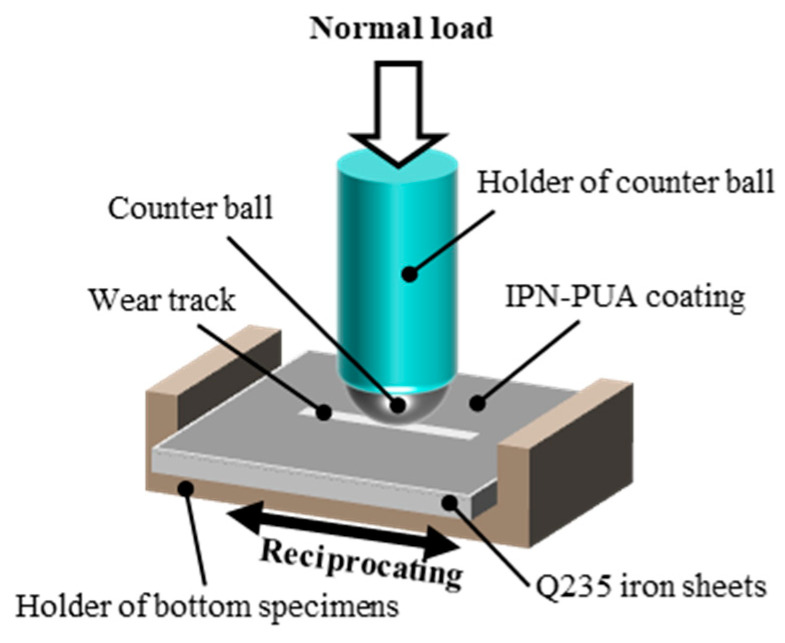
Schematic illustration of the friction apparatus.

The friction tests were conducted with a CSM tribometer (Anton Paar, Graz, Austria). The working parameters of the friction tests were configured as follows: a load of 5 N, a reciprocating frequency of 2 Hz, and a reciprocating stroke of 8 mm. The counter ball was a 6 mm sphere fabricated from GCr15 bearing steel. The graphical depiction of the friction apparatus is presented in Figure 3.

A white-light confocal scanner (Anton Paar, Graz, Austria) furnished with an optical microscope was utilized to obtain the surface morphology of the coating. The optical microscope therein was utilized to scrutinize the morphology of the coating wear track. Subsequently, a series of post-processing operations were implemented through Image Plus software (version of 2.19), facilitating the obtainment of the three-dimensional topography and roughness of the coating surface and the cross-sectional curves of the wear trace.

An SEM (scanning electron microscope, ProX, Phenom, Eindhoven, The Netherlands) outfitted with an Energy Dispersive Spectrometer (EDS) was employed to scrutinize the morphology of the solid lubricant particles, the surface topography of the coating surface, and the microscopic topography of the wear trace.

A nanoindentation tester (Anton Paar, NTH^2^, Switzerland) was used to test the nano-hardness and nano-modulus of the coatings. The loading force was 5 mN and was maintained for 10 s with the loading and unloading speed of 10 mN/min.

In a 40 °C water bath environment, the kinematic viscosity of the paints was measured using a Pine Viscometer following the GB/T 265-1988 standard [44]. For IPDI-HPA and IPDI-HEA, a 1.2 mm diameter capillary viscometer (constant of 0.0945 mm^2^/s^2^) was used. For TDI-HPA and TDI-HEA, a 2.0 mm diameter capillary viscometer (constant of 0.749 mm^2^/s^2^) was employed. The detailed viscosity measurement results are presented in Table 1. It can be found that the paints with the IPDI component have relatively low kinematic viscosities, and the differences among them are also small. In contrast, the kinematic viscosities of paints with the TDI component increase significantly. The viscosity of TDI-HEA exceeds 1000 mm^2^/s, indicating that the leveling property on a flat surface will be greatly affected.

## 3. Results and Discussion

### 3.1. Properties of IPN-PUA Coatings with Diverse Components

#### 3.1.1. Surface Morphologies of IPN-PUA Coatings

**Figure 4:**
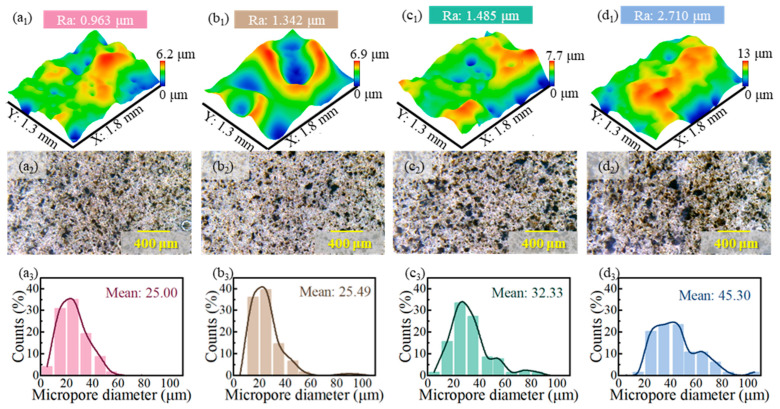
Surface morphologies and roughness of IPN-PUA coatings: surface morphologies and roughness of the coatings surface (**a_1_**) IPDI-HPA, (**b_1_**) IPDI-HEA, (**c_1_**) TDI-HPA, (**d_1_**) TDI-HEA; microstructure of the coating surface: (**a_2_**) IPDI-HPA, (**b_2_**) IPDI-HEA, (**c_2_**) TDI-HPA, (**d_2_**) TDI-HEA; statistical results of micropores diameter (**a_3_**) IPDI-HPA, (**b_3_**) IPDI-HEA, (**c_3_**) TDI-HPA, (**d_3_**) TDI-HEA.

The surface morphologies of the IPN-PUA coatings with diverse components were acquired using a white-light confocal microscope and an optical microscope, as presented in Figure 4. Subsequently, the roughness of the coatings surface, calculated with Image Plus software, is displayed in Figure 4a_1_–d_1_. The roughness measurements suggest that the coating surfaces comprising the IPDI and HPA components are smoother, whereas the surfaces of the coatings with the TDI or HEA components are rougher. This phenomenon may potentially result from the growth in the stickiness of the paints containing TDI or HEA components, and the fluidity worsens, thereby leading to a larger surface roughness of the coating [45,46]. The microstructure of the coating surface is depicted in Figure 4a_2_–d_2_, highlighting the presence of micropores on the surface of the coatings. The statistical results regarding the diameter of these micropores are shown in Figure 4a_3_–d_3_. It can be observed that coatings with TDI or HEA components feature larger pore sizes. Notably, the surface of the TDI-HEA coating exhibits the largest micropores, as illustrated in Figure 4d_2_.

#### 3.1.2. Components of IPN-PUA Coatings

**Figure 5:**
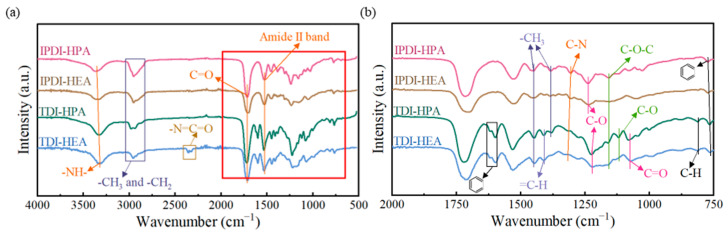
FTIR of IPN-PUA coatings. (**a**) Full spectrum and (**b**) enlarged image.

The Fourier transform infrared spectroscopy (FTIR) of the coatings is illustrated in Figure 5.

It becomes evident that the infrared spectra of the IPDI-HPA and IPDI-HEA coatings exhibit a high degree of similarity. The stretching vibration of the -NH- group (3340 cm^−1^), C=O stretching vibration (1709 cm^−1^), and amide II band (1527 cm^−1^, combination of N-H bending and C-N stretching), as well as the C-N stretching vibration band (1304 cm^−1^) provide evidence for the urethane group in the coating. The relatively lower wavenumber of the C=O stretching vibration indicates the formation of hydrogen bonds. Within 2830–3024 cm^−1^, symmetric stretching vibration bands of -CH_3_ and -CH_2_ groups are detected. The -CH_3_ asymmetric bending band is at 1451 cm^−1^ with its symmetric stretching band at 1384 cm^−1^, mainly due to the methyl and methylene present in MMA. The band at 1238 cm^−1^ represents C-O stretching in the ester groups of HPA or HEA and MMA; at 1156 cm^−1^ is the stretching vibration of the ether (C-O-C) bond. Meanwhile, the band at 766 cm^−1^ is likely to have originated from the out-of-plane bending vibration of C-H bonds on the benzene ring in GO. Within the spectral range of 2830–3024 cm^−1^, symmetric stretching vibration bands corresponding to -CH_3_ and -CH_2_ groups can be detected. Specifically, the -CH_3_ asymmetric bending band emerges at 1451 cm^−1^, while its symmetric stretching band is located at 1384 cm^−1^. These bands are primarily generated by the methyl and methylene groups in MMA.

When the component of the coating was changed from IPDI to TDI, significant changes occurred in the FTIR spectrum. A small number of characteristic bands of the asymmetric stretching vibration of -N=C=O appeared at 2346 cm^−1^ in the TDI-HEA coating, indicating that a small amount of TDI remained unreacted. The bending vibration band of =C-H appeared at 1410 cm^−1^, suggesting that due to the increased viscosity, the C=C had not fully reacted during the curing process [47]. Moreover, characteristic bands of the benzene ring (at 1617 cm^−1^), C-O stretching vibration of the ether bond on the benzene ring (at 1115 cm^−1^), and the out-of-plane bending vibration of C-H in the benzene ring (at 810 cm^−1^) emerged, all of which corresponded to the existence of TDI. Moreover, due to the presence of the benzene ring, conjugation occurred between the ester group and the benzene ring, resulting in the appearance of the C=O group (at 1115 cm^−1^) and the shift of the C-O stretching vibration to 1224 cm^−1^.

#### 3.1.3. Friction Properties of IPN-PUA Coatings

**Figure 6:**
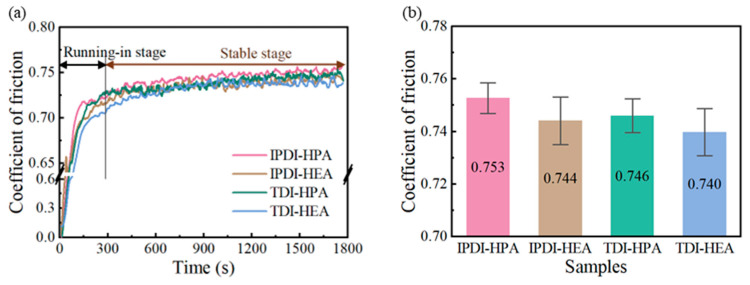
Coefficients of friction of IPN-PUA coatings with different components: (**a**) trend of COF variation with time, (**b**) average COF.

The friction coefficients of the IPN-PUA coatings with varying components are presented in Figure 6a,b. There are obvious running-in and stable stages for the coatings during the friction process, as shown in Figure 6a. Under the circumstance where no lubricating phase is present, it is evident that the friction coefficients of these IPN-PUA coatings are relatively close, all stabilizing within the range of 0.74 to 0.76. Specifically, the COF of the coating that contains TDI is marginally lower than that with IPDI as a component. At the same time, the COF of the HEA coating is also slightly lower compared to that of the HPA coating.

**Figure 7:**
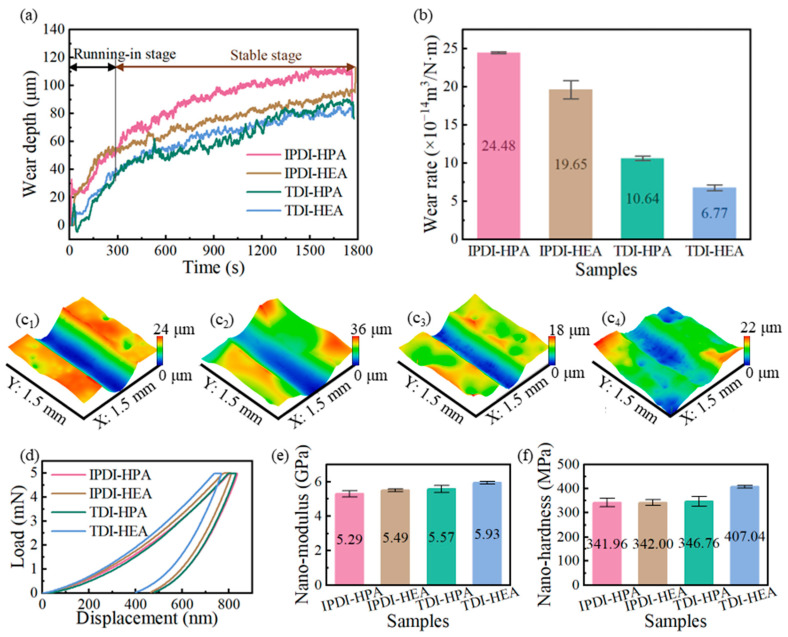
Wear of IPN-PUA coatings with different components: (**a**) trend of online wear depth variation with time, (**b**) average offline wear rate; three-dimensional morphologies of the wear tracks: (**c_1_**) IPDI-HPA, (**c_2_**) IPDI-HEA, (**c_3_**) TDI-HPA, (**c_4_**) TDI-HEA. Nano-mechanical properties of IPN-PUA coatings with different components: (**d**) typical displacement–load curves in nanoindentation, (**e**) nano-modulus, and (**f**) nano-hardness.

The wear performances of IPN-PUA coatings with varying components are exhibited in Figure 7a,b. As delineated in Figure 7a, the variation trend of the online wear depth with respect to the friction duration can be observed. The running-in stage and stable stage in Figure 7a are distinguished based on the stages in Figure 6a. It is evident that the IPDI coating demonstrates a larger wear depth during the running-in stage. Concurrently, within the stable stage, the slope of the online wear depth for the HPA coating is slightly steeper than the HEA coating. These manifestations imply that the coatings composed of IPDI and HPA components possess poorer wear resistance and undergo greater deformation under load.

The offline wear topography after the frictional process was examined utilizing a white-light confocal microscope, as portrayed in Figure 7c_1_–c_4_. The offline wear rate, computed by means of the wear rate computational formula, is presented in Figure 7b. It can be noted that the TDI and HEA combinations exhibit a lower wear rate in comparison to the IPDI and HPA ones, with TDI having a more conspicuous impact on the wear rate. Specifically, the wear rate of the TDI-HPA coating is 10.64 × 10^−14^ m^3^/N·m, signifying a 56.5% diminution in comparison to that of IPDI-HPA. The nano-mechanical properties of the coatings are depicted in Figure 7d,f. It is observed that the higher the nano-modulus and nano-hardness of the coating, the lower is the wear rate of the coatings. This can be ascribed to the presence of a benzene ring in the molecular architecture of TDI [48], which confers greater rigidity than the isophorone ring in IPDI. Consequently, the stiffness of the molecular chains within the coating is augmented, further elevating the hardness and modulus of the coating [49]. The augmented hardness and modulus of the coating markedly curtail the wear rate [50]. In contrast, the wear rate of IPDI-HEA is only 19.7% lower than that of IPDI-HPA. This is predominantly due to the fact that the HEA molecular chain is shorter than that of HPA, curtailing the flexibility of the molecular chains and concomitantly enhancing the hardness and modulus of the coatings, thereby precipitating a reduction in the wear rate.

In summation, the impact of the benzene ring in TDI on the wear rate preponderates over that of the short molecular chain of HEA to a significant extent.

#### 3.1.4. Wear Morphologies of IPN-PUA Coatings

The three-dimensional morphology of the wear scar is illustrated in Figure 7c. It is observable that as the coating components progressively transform into TDI and HEA, the surfaces of the coating flanking the wear scar become increasingly uneven, which is in line with the morphology of the coating surface depicted in Figure 4.

**Figure 8:**
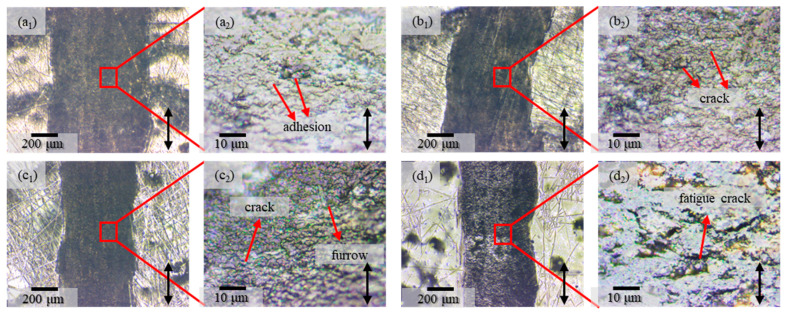
Microscopic optical morphologies of the coatings after friction. Morphologies of the wear tracks: (**a_1_**,**a_2_**) IPDI-HPA, (**b_1_**,**b_2_**) IPDI-HEA, (**c_1_**,**c_2_**) TDI-HPA, (**d_1_**,**d_2_**) TDI-HEA. The direction of the arrows indicates the relative frictional movement direction.

The surface morphology of the wear scar is demonstrated in Figure 8a_1_–d_2_. Notably, the variation trend of the wear scar width coincides with that of the wear rate exhibited in Figure 7b. By analyzing the locally amplified morphology within the wear scar, it can be ascertained that the predominant wear mode of the IPDI-HPA coating is adhesive wear [3], as manifested in Figure 8a_2_. In contrast, minute failure cracks emerge inside the wear scars of the IPDI-HEA and TDI-HPA coatings, as presented in Figure 8b_2_,c_2_. Concurrently, in the TDI-HPA coating, a sparse number of furrows come into sight, signifying the incipient emergence of fatigue wear. The wear morphology of the TDI-HEA coating reveals a profusion of fatigue cracks, as indicated in Figure 8d_2_.

In summary, during the course wherein the coating components gradually transition from IPDI and HPA to TDI and HEA, the wear mode of the coating undergoes a progressive shift from adhesive wear to fatigue wear. This transformation is attributable to the augmented hardness and modulus of the coating. Owing to the appearance of fatigue wear, the COF of the coating exhibits a marginal decline.

#### 3.1.5. Comprehensive Performance of IPN-PUA Coatings with Diverse Components

**Figure 9:**
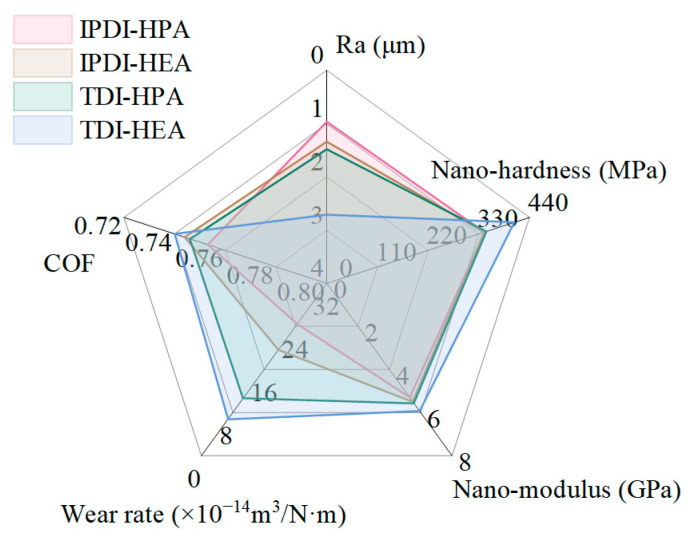
Radar chart of the comprehensive performance of surface roughness, nano-hardness, nano-modulus, COF, and wear rate of IPN-PUA coatings with diverse components.

According to the experimental findings, the radar chart of the comprehensive performance of the surface roughness, COF, and wear rate of IPN-PUA coatings with diverse components is presented in Figure 9. The smaller the values of these indicators, the better is the corresponding performance. In the radar chart illustrated in Figure 9, a larger area of the shaded part represents a better comprehensive performance of the material.

The COFs of the four coatings display a relatively high degree of similarity. Among them, the IPDI-HPA coating exhibits the lowest surface roughness; nevertheless, it suffers from the largest wear rate. On the other hand, the TDI-HEA coating presents the smallest wear rate and the largest nano-hardness and nano-modulus, yet its surface roughness is the worst. Consequently, although the performance of IPDI-HPA and TDI-HEA stands out in mechanical properties and wear resistance, their comprehensive performance is nevertheless far from satisfactory because of their high COF [51,52].

Overall, it is observed that the shaded area corresponding to the TDI-HPA coating is the largest, which indicates its possession of the best comprehensive performance. However, it should be noted that the friction coefficient of IPN-PUA coatings remains at a relatively elevated level. Therefore, the introduction of a lubricating phase is deemed necessary to enhance its tribological performance.

### 3.2. IPN-PUA Coatings with Different Lubricating Phases

Utilizing the TDI-HPA coating, which exhibits the best comprehensive performance, as the matrix material, modifications were made to the tribological properties of the coating by incorporating graphite, PTFE particles with a size of 25 μm, and PTFE particles with a size of 75 μm as lubricating phases. It has been demonstrated that an insufficient lubricant content leads to inadequate lubrication, whereas an excessive lubricant content significantly compromises the mechanical properties of the matrix, paradoxically increasing the wear rate due to the weakened structural integrity [53,54]. Therefore, the mass ratio of the lubricating phase to the matrix was set at 1:10. The three resultant coatings were designated as T-P-G, T-P-25F, and T-P-75F, respectively.

#### 3.2.1. Surface Morphologies of the Self-Lubricating Coatings

**Figure 10:**
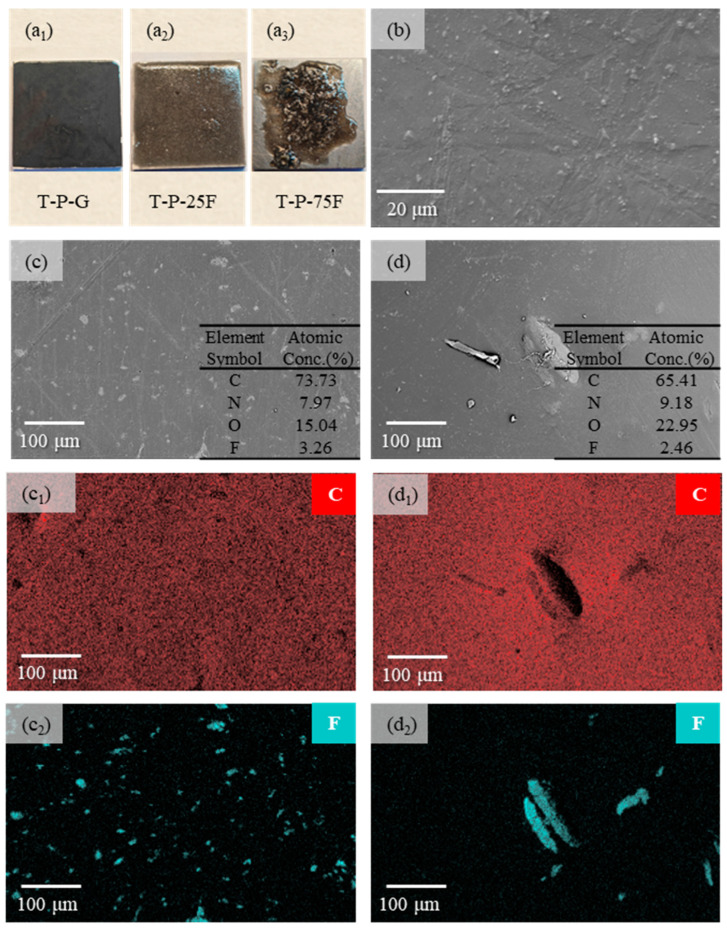
Surface morphology of graphite- and PTFE-modified coatings. Photographs of the three composite coatings after UV curing: (**a_1_**) T-P-G, (**a_2_**) T-P-25F, and (**a_3_**) T-P-75F. The SEM images of the morphologies and the EDS elemental distributions of self-lubricating surfaces: (**b**) T-P-G, (**c**) T-P-25F, and (**d**) T-P-75F. (**c_1_**) and (**c_2_**) are the distributions of the C and F elements corresponding to (**c**), respectively. (**d_1_**) and (**d_2_**) are the distributions of C and F elements corresponding to (**d**), respectively.

Photographs of the three composite coatings after UV curing are displayed in Figure 10a_1_–a_3_, respectively. The surface of the T-P-G coating is remarkably smooth, and the surface of the T-P-25F coating shows a few protrusions and holes. For the T-P-75F coating, the large particle size of PTFE causes the viscosity of the premixed polymer to rise after mixing, so much so that it is unable to cover the entire iron sheet. At the same time, it can be observed that there is macroscopic phase separation between the matrix phase (TDI-HPA) and the lubricating phase (PTFE) [55,56], as depicted in Figure 10a_3_. Measurements were carried out to determine the thicknesses of the coatings. The T-P-G coating has a thickness ranging from 0.16 mm to 0.17 mm, while the T-P-25F coating measures 0.34 mm to 0.35 mm in thickness, and the T-P-75F coating has a thickness of ~0.36 mm to 0.37 mm.

The SEM images of the morphologies and the EDS elemental distributions of the self-lubricating surfaces are presented in Figure 10b–d. As illustrated in Figure 10b, the light-colored areas of the SEM image of the T-P-G coating are flake-like graphite, which is similar to the morphology of graphite shown in Figure 2. The T-P-G coating surface is smooth, and the graphite is uniformly distributed on the coating surface. Through EDS analysis, it is found that carbon, oxygen, nitrogen, and fluorine elements exist on the surfaces of both the T-P-25F and T-P-75F coatings. Based on the combined analysis of EDS and SEM, it can be determined that the light-colored regions in Figure 10c,d are PTFE. As displayed in Figure 10d, PTFE is uniformly distributed on the T-P-25F coating surface. However, due to the macroscopic phase separation that occurred between the PTFE on the surface of the T-P-75F coating and the matrix, the PTFE in Figure 10d also exhibits an obvious agglomeration phenomenon.

#### 3.2.2. Friction Performance of Self-Lubricating Coatings

**Figure 11:**
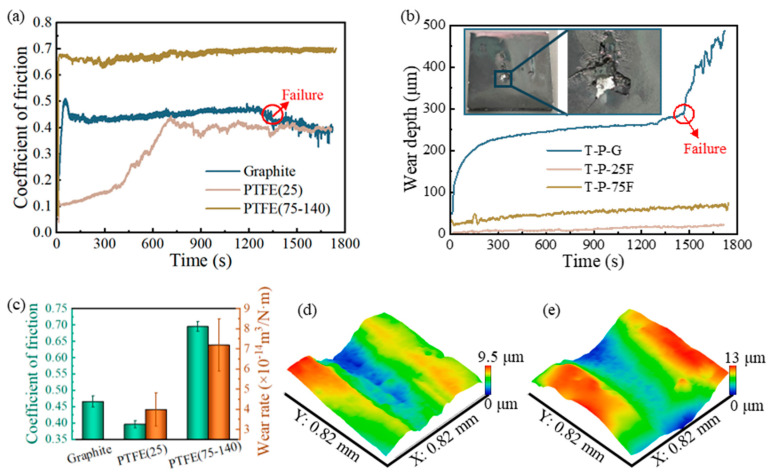
Friction performance of IPN-PUA coatings with different lubricating phases. The curves of (**a**) COF and (**b**) online wear depth varying over time. (**c**) Average COF and wear rate. 3D morphology of wear scars: (**d**) T-P-25F, (**e**) T-P-75F.

The curves of the COF and online wear depth varying over time for the coatings containing different lubricating phases are presented in Figure 11a,b.

For the T-P-G coating, after a very short running-in stage, its friction coefficient stabilized at around 0.465. Its online wear depth also tends to stabilize after the running-in stage. However, when the friction time reaches approximately 1457 s, the coating begins to fail, with its friction coefficient dropping rapidly and the wear rate rising sharply. In Figure 11b, the photographs of the T-P-G coating before and after wear failure are shown. It can be perceived that after friction failure, the surface layer of the coating fractures, and there is still uncured premixed polymer beneath the coating. That is to say, after being cured by UV light, the T-P-G coating has a cured layer on its surface with a thickness of only about 250 μm, and beneath this cured layer, there is another layer approximately 250 μm thick that has not yet been cured. Notably, the thickness of the uncured T-P-G coating is less than that of the cured T-P-25F and T-P-75F coatings under the same curing duration and process conditions. The fact that the coatings with a greater thickness are fully cured while the thinner T-P-G coating is not completely cured indicates that thickness is not a factor influencing the curing of the coating. This might be because of the conjugated π-bond system of graphite, which is capable of absorbing the energy of ultraviolet photons and converting it into thermal energy or other forms of energy, thus preventing the direct penetration of ultraviolet lights [57,58]. Moreover, the black property of graphite [59] causes the light transmittance of the coating surface after molding to drop dramatically, making it more difficult for UV light to penetrate the cured layer. When the wear depth reaches the thickness of the cured layer, the coating fails and enters the stage of rapid wear. Interestingly, after failure, the friction coefficient starts to decrease. This may be because the uncured premixed polymer enters the interface, changing the dry friction to liquid lubricating with graphite and thus reducing the friction coefficient.

In Figure 11b, only the T-P-G coating underwent significant deformation within the initial 150 s of the friction experiment. This phenomenon further confirms its incomplete cured state. In addition, there was no significant deformation in T-P-25F and T-P-75F. Instead, they were worn out at a constant speed, which indicates that T-P-25F and T-P-75F have been completely solidified.

Although both the T-P-25F coating and the T-P-75F coating contain PTFE, there is a huge difference in their demonstrated frictional properties. The 25 μm sized PTFE particles significantly reduce the COF of the coating, lowering the friction coefficient of the TDI-HPA coating from 0.746 to 0.395, which represents a reduction of approximately 47%. In contrast, PTFE with a particle size in the range of 75–140 μm has a minimal impact on the COF, merely reducing it to 0.695, which is only a 6.8% decrease. This may be due to the obvious phase separation that occurs between the PTFE with a particle size of 75–140 μm and the matrix, making it difficult for the PTFE to provide a lubricating effect during the friction process. At the same time, the roughness of the T-P-75F coating surface increases, exacerbating the vibration during the friction process. Within the T-P-25F coating, the PTFE and the matrix are distributed in a uniform manner. During the friction process, the self-lubricating property inherent to the PTFE gradually comes to the fore, bringing about a substantial reduction in COF. In addition, the online wear rate of the T-P-25F is also lower than the T-P-75F coating. The friction and wear test outcomes showed that the PTFE particles with a diameter of 25 μm significantly outperform those with a diameter of 75–140 μm in terms of enhancing the tribological characteristics of the coating.

This phenomenon differs from the influence law of different PTFE particle sizes on epoxy resin-based bulk composites [35]. In this research, it has been observed that the larger the PTFE particle size, the lower are the COF and wear rate of the composite material. This may be due to the fact that the state of the epoxy prepolymer in this study is “paste-like”, which is more viscous than the TDI-HPA paints, so that even if the particle size of the PTFE becomes larger, it can still be uniformly distributed and not undergo obvious phase separation. The results indicate that there are differences in the influence law of the particle size of solid lubricants on the tribological properties of composite materials between bulk materials and coating materials.

The average COF and wear rate of self-lubricating coatings containing different lubricating phases are presented in Figure 11c. Due to the failure of the T-P-G coating, it proved infeasible to acquire the three-dimensional morphology of its wear scar. The three-dimensional morphologies of the wear tracks of the T-P-25F coating and the T-P-75F coating are illustrated in Figure 11d,e. The calculated wear rate of the T-P-25F coating was 3.97 × 10^−14^ m^3^/N·m, which was approximately 62.7% lower than that of the pure TDI-HPA coating (10.64 × 10^−14^ m^3^/N·m), demonstrating excellent wear resistance. Although the COF of the T-P-25F coating is higher than that of the polymer coatings in References [3,4,5,6,7,8,9], it exhibits excellent wear resistance.

#### 3.2.3. Wear Morphology of Self-Lubricating Coatings

**Figure 12:**
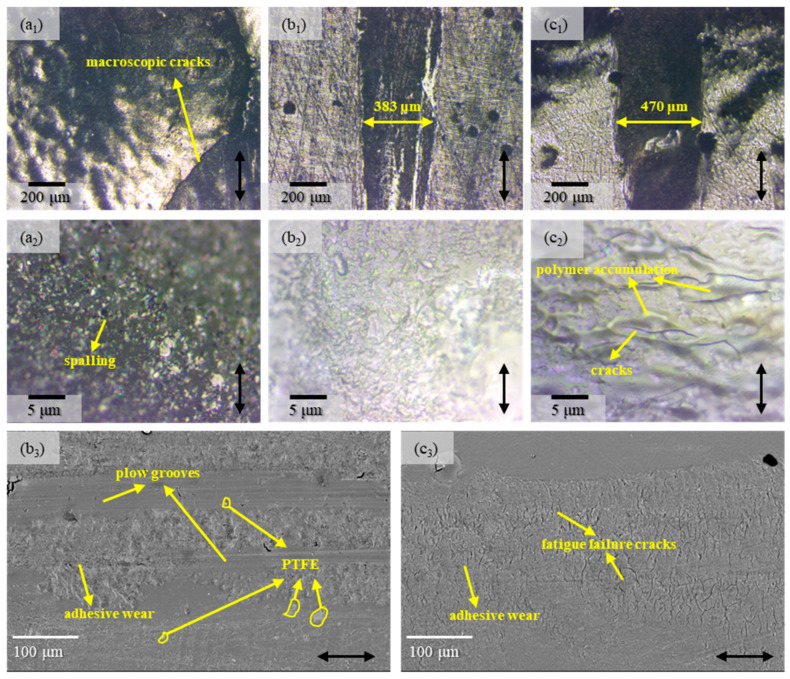
The microscopic morphologies of the wear tracks of the coatings with different lubricating phases: (**a_1_**,**a_2_**) T-P-G, (**b_1_**–**b_3_**)T-P-25F, (**c_1_**–**c_3_**) T-P-75F. The direction of the arrows indicates the relative frictional movement direction.

The microscopic morphologies of the wear tracks of coatings with different lubricating phases are presented in Figure 12.

Owing to the premature failure of the T-P-G coating, it was extremely challenging to discern the width of its wear scar. As shown in Figure 12a_1_, macroscopic cracks manifested within the wear tracks. Nevertheless, from the locally enlarged view in Figure 12a_2_, it could be observed that some spalling had also taken place during the wear process.

Through the optical microscope images shown in Figure 12b_1_–b_3,_c_1_–c_3_, it can be discerned that the wear scar width of the T-P-25F coating is markedly narrower than that of the T-P-75F coating. Simultaneously, on either side of the wear scar, the roughness of the T-P-25F coating is conspicuously lower than that of the T-P-75F coating. Upon inspecting the locally enlarged view of the wear scar, it becomes evident that the surface of the wear track of the T-P-25F coating is considerably smoother, whereas the surface of the T-P-75F wear track displays cracks and polymer accumulation around the cracks.

To further explore the wear mechanisms of the T-P-25F and T-P-75F coatings, SEM characterization was conducted, with the results depicted in Figure 12b_3_,c_3_. It could be evidently perceived that conspicuous plow grooves were present on the surface of the T-P-25F. Remarkably, the existence of PTFE was discerned within the wear track, and adhesive wear was also noticed. The presence of PTFE mitigated the adhesive wear of the material, consequently reducing the wear rate. Owing to the generation of the PTFE transfer film, the friction coefficient of the material was diminished. Conversely, PTFE was not detected on the surface of the T-P-75F wear track, which further corroborates that PTFE was distributed extremely unevenly on the coating surface. Due to the absence of the lubricating effect furnished by PTFE, copious fatigue failure cracks sprang up on the coating surface, which resembled the wear morphology of the TDI-HPA coating without PTFE (Figure 8c_2_).

In sum, the PTFE particles with small diameters can be evenly dispersed across the coating surface, thereby furnishing a steady and efficient lubricating effect during the friction process. By contrast, the PTFE particles with large diameters evidently tend to aggregate within the coating, exerting scarcely any influence on enhancing the friction performance of the coating. The T-P-25F coating exhibits outstanding wear resistance, which makes it a promising candidate for application as a wear-resistant coating in joint bearings.

## 4. Conclusions

This research comprehensively explored the properties of UV-cured IPN-PUA self-lubricating coatings regarding matrix constituents and lubricants, which offers a fundamental understanding of the factors that impact the performance of UV-cured IPN-PUA self-lubricating coatings. The coatings have promising applications as wear-resistant coatings in joint bearings because of their excellent wear resistance and easy moldability. However, their performance in complex environments remains poorly understood. To address this, future research could concentrate on investigating the long-term stability of these coatings in such complex environments. The specific conclusions are as follows:

- It was determined that the selection of isocyanate and hydroxyl acrylate monomers had a significant influence on the coating properties. Generally, TDI-based coatings demonstrated better wear resistance in comparison to IPDI-based ones, and coatings containing HEA had lower wear rates than those containing HPA.
- Among the four basic coatings, TDI-HPA exhibited the most favorable comprehensive performance, with its surface roughness of 1.485 μm and wear rate of 10.64 × 10^−14^ m^3^/N·m with a 56.5% reduction compared to IPDI-HPA. However, due to its high COF of 0.746, its overall performance still failed to reach a satisfactory level.
- In coatings, 25 μm sized PTFE particles outperform their 75 μm sized counterparts in terms of anti-friction and wear-resistance capabilities. This phenomenon is different from the impact pattern of particle size on the tribological characteristics in bulk materials. Small-sized PTFE particles are capable of achieving a more homogeneous dispersion within the coating matrix. In contrast, large-sized PTFE particles tend to induce phase separation and exert a negligible influence on enhancing the tribological properties.
- The T-P-25F coating showed a certain self-lubricating performance and remarkable wear-resistance performance. Its COF and wear rate were 0.395 and 3.97 × 10^−14^ m^3^/N·m, respectively, representing a reduction of 47% and 62.7% compared to the pure TDI-HPA coating.
- Graphite also had an impact on the friction performance of the coating. However, its light-blocking property hindered the formation of the coating during UV curing.

## Figures and Tables

**Table 1:** Viscosity of paints.

Paint	IPDI-HPA	IPDI-HEA	TDI-HPA	TDI-HEA
Viscosity (mm^2^/s)	22.02 22.73	24.31 25.08	106.03 94.79	1127.87 1176.06
Viscometer capillary diameter	1.2 mm	1.2 mm	2 mm	2 mm

## Data Availability

The data presented in this study are available on request from the corresponding author. The data are not publicly available due to privacy restrictions.
